# Cooperative Emissions Trading Game: International Permit Market Dominated by Buyers

**DOI:** 10.1371/journal.pone.0132272

**Published:** 2015-08-05

**Authors:** Keita Honjo

**Affiliations:** Center for Social and Environmental Systems Research, National Institute for Environmental Studies, Tsukuba, Ibaraki, Japan; University of Florida, UNITED STATES

## Abstract

Rapid reduction of anthropogenic greenhouse gas emissions is required to mitigate disastrous impacts of climate change. The Kyoto Protocol introduced international emissions trading (IET) to accelerate the reduction of carbon dioxide (CO_2_) emissions. The IET controls CO_2_ emissions through the allocation of marketable emission permits to sovereign countries. The costs for acquiring additional permits provide buyers with an incentive to reduce their CO_2_ emissions. However, permit price has declined to a low level during the first commitment period (CP1). The downward trend in permit price is attributed to deficiencies of the Kyoto Protocol: weak compliance enforcement, the generous allocation of permits to transition economies (hot air), and the withdrawal of the US. These deficiencies created a buyer’s market dominated by price-making buyers. In this paper, I develop a coalitional game of the IET, and demonstrate that permit buyers have dominant bargaining power. In my model, called cooperative emissions trading (CET) game, a buyer purchases permits from sellers only if the buyer forms a coalition with the sellers. Permit price is determined by bargaining among the coalition members. I evaluated the demand-side and supply-side bargaining power (DBP and SBP) using Shapley value, and obtained the following results: (1) Permit price is given by the product of the buyer’s willingness-to-pay and the SBP (= 1 − DBP). (2) The DBP is greater than or equal to the SBP. These results indicate that buyers can suppress permit price to low levels through bargaining. The deficiencies of the Kyoto Protocol enhance the DBP, and contribute to the demand-side dominance in the international permit market.

## Introduction

Scientific evidence clearly indicates that climate change is driven by greenhouse gases (mainly carbon dioxide, CO_2_) emitted from human activities [1]. Rapid reduction of anthropogenic CO_2_ emissions is required to protect present and future generations from disastrous impacts of climate change [2–4]. The United Nations Framework Convention on Climate Change (UNFCCC) agreed the Kyoto Protocol in 1997, and decided to reduce CO_2_ emissions from Annex B Parties to the levels in 1990 [5, 6]. The Kyoto Protocol introduced international emissions trading (IET) to accelerate the emission reduction. Each country owns marketable emission permits equivalent to its emission cap specified in the Kyoto Protocol. Countries facing permit shortfalls need to purchase permits from other countries. The costs for acquiring additional permits provide buyers with an incentive to reduce their CO_2_ emissions. According to the basic competitive model in economics, emissions trading is a cost-effective way of controlling CO_2_ emissions [7–11].

The Kyoto Protocol completed the first commitment period (CP1) in 2012. The international permit market under CP1 was far from an efficient market drawn by the basic competitive model. The annual average price of permits (assigned amount units, AAUs) peaked at US$12.92 per ton of CO_2_ in 2009, and then rapidly declined to US$6.77 in 2011 [12–14]. Even much lower prices were reported in 2012 [15]. These prices are relatively low compared to many estimates of the social cost of carbon (SCC, marginal damage caused by an additional ton of CO_2_ emissions) [16, 17]. A permit price lower than the SCC means the underestimation of the risk of climate change. As buyers are allowed to acquire additional permits at low costs, the emission reduction would not reach the optimal level.

The downward trend in permit price is attributed to deficiencies of the Kyoto Protocol, which have been discussed by many authors. First, the Kyoto Protocol has no strict sanctions against non-participation and non-compliance [18, 19]. In Marrakesh Accords, the Kyoto Protocol introduced some non-financial sanctions against non-compliance [20]. However, a non-compliant country can avoid the sanctions by withdrawing from the Kyoto Protocol. The Kyoto Protocol depending on voluntary actions of the members is vulnerable to free riding. If a buyer loses willingness to purchase permits, it can withdraw from the market without paying costs. In fact, the IET has experienced the withdrawal of the top-three buyers: the US, Japan, and Canada [21–23]. The absence of major buyers leads to a smaller demand and a lower price. Moreover, the right of buyers to withdraw from the market is a threat to sellers. If buyers exercise the right, sellers cannot gain benefits from emissions trading. Therefore sellers are forced to provide permits at low prices which are acceptable to buyers.

**Fig 1:**
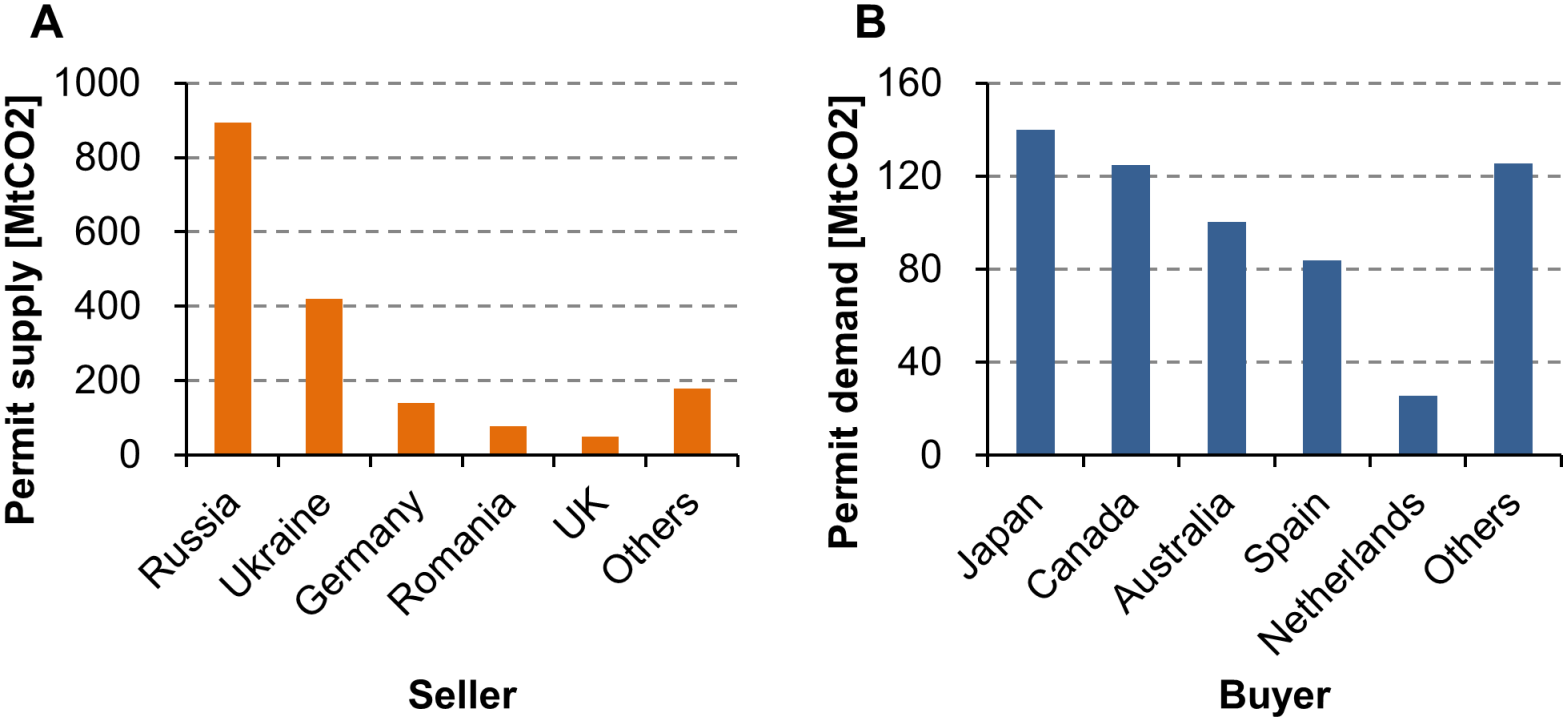
Permit supply and demand in Annex B Parties (annual average, 2008–2012). Calculated from CO_2_ emission data [24] and the Quantified Emission Limitation or Reduction Commitment (QELRC) of the Kyoto Protocol [5]. The US and the EU bubble are not included. Let *G* be annual average emissions from a country between 2008 and 2012, and let *C* be the emission cap (per year) of the country specified in the Kyoto Protocol. (A) If *G* < *C*, the country is a seller with a supply of *C* − *G*. (B) If *C* < *G*, the country is a buyer with a demand of *G* − *C*.

**Fig 2:**
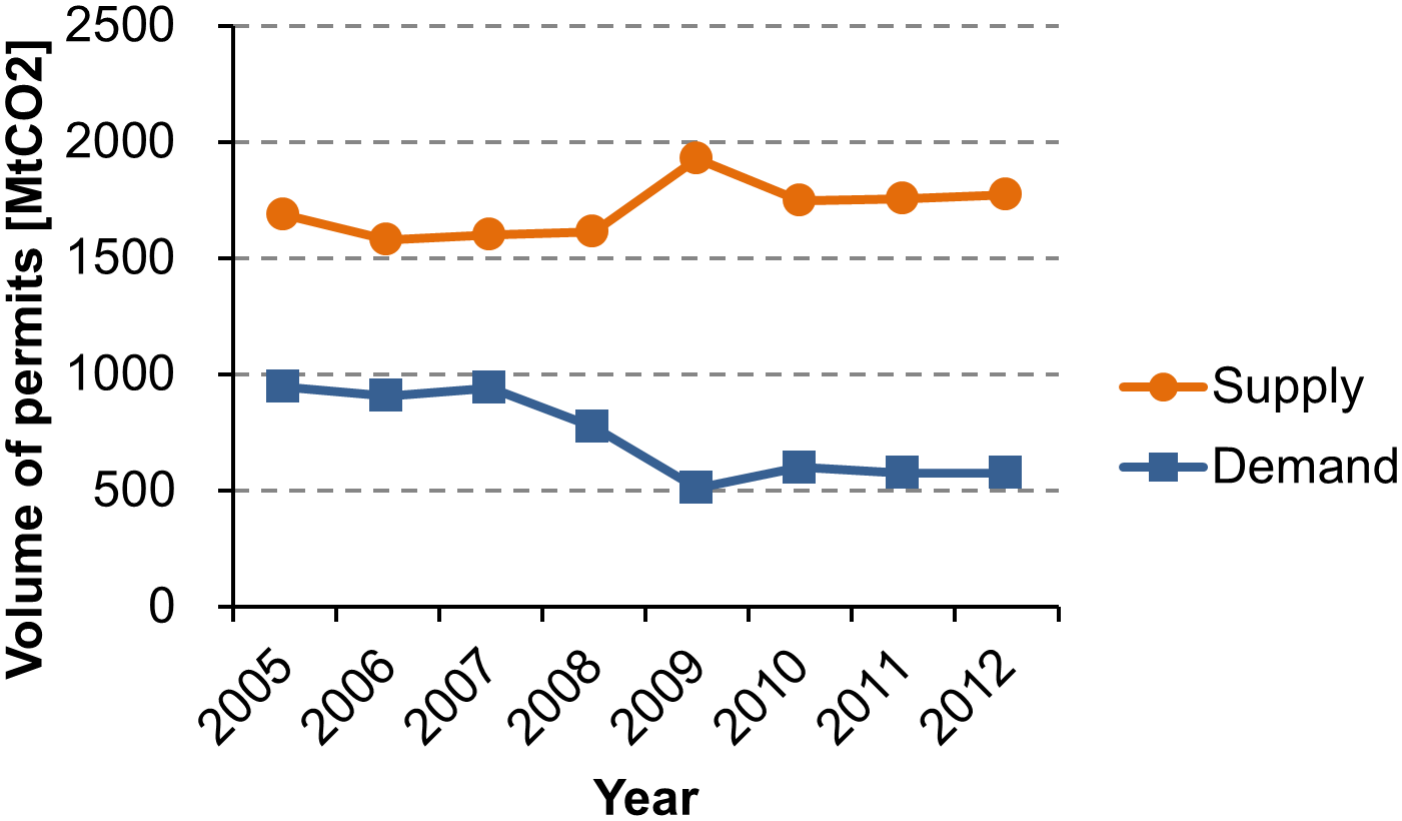
Aggregate permit supply and demand in Annex B Parties, 2005–2012. Calculated from CO_2_ emission data [24] and the QELRC of the Kyoto Protocol [5]. The US is not included.

Second, the allocation of permits to transition economies (specifically Russia and Ukraine) was too generous [6, 11]. Due to the hot air, the IET under CP1 has suffered from the oversupply of permits (Fig 1). This trend was accelerated by the withdrawal of the US from the Kyoto Protocol. If the US participated in the IET, it would have been the largest buyer with a demand of 876 MtCO_2_ per year [24]. Several authors estimate the impacts of the US withdrawal on permit price using computational models [25–30]. The 2008–2009 global financial crisis widened the supply-demand gap (Fig 2). In 2009, CO_2_ emissions from Annex B Parties decreased in response to the economic downturn [24]. As a result, the demand-side permit shortfall decreased, while the supply-side permit surplus increased. The vulnerable market has been sustained by Japan’s purchasing power [12–15].

These observations suggest that the international permit market under CP1 was a buyer’s market. In a buyer’s market, at least one buyer exercises market power to manipulate price. The cost-effective performance of emissions trading assumes a competitive market formed by price takers [31]. If there exists a price maker in the market, emissions trading may fail to achieve the optimal price [32] (see [9] for the extensions of this result). In the context of the IET under CP1, many authors have investigated market power of sellers, especially transition economies. By extending Hahn’s model [32], Maeda [33] concludes that sellers can exercise market power by forming a cartel but buyers cannot. The impacts of seller cartels on permit price are estimated by computational models [26–28, 30]. Meanwhile, Godal and Meland [34] demonstrate that a seller cartel is not a dominant strategy for sellers. Strategic behavior of buyers can nullify benefits from a seller cartel. They also show that a coalition containing both buyers and sellers is profitable. In the IET, permit price is determined by bargaining. Power relationships between buyers and sellers play the important role in price formation. Further research on market power is necessary to understand the IET.

Game theory is a useful tool to solve bargaining problems. There are two types of approaches: non-cooperative and cooperative. Non-cooperative games focus on how self-interest players form a stable coalition. Strategic behavior of players is described by solution concepts satisfying individual rationality (e.g. max-min solution and Nash equilibrium). The non-cooperative approach has contributed to the analysis of international climate negotiations [35–43]. Results of non-cooperative games suggest that the grand coalition containing all countries is unlikely to be stable because of free riding. In contrast, cooperative games (coalitional games) focus on how benefits of a coalition are allocated to the members [44–46]. A payoff allocation is determined by solution concepts satisfying both individual and social rationality (e.g. core and Shapley value). Solution concepts for coalitional games are associated with bargaining power of players [47, 48]. The cooperative approach has been applied to the allocation of natural and environmental resources [49–52]. Several authors have discussed non-emptiness of the core of economies with externalities [42, 53–56]. A non-empty core implies that a stable and efficient payoff allocation is possible.

In this paper, I develop a coalitional game of the IET, and demonstrate that buyers have dominant bargaining power. In my model, called cooperative emissions trading (CET) game, a buyer purchases permits from sellers only if the buyer forms a coalition with the sellers. In the real market, buyers may cooperate with each other. For simplicity, however, coalitions containing more than one buyer (multi-buyer coalitions) are excluded. This paper focuses on bargaining between each buyer and sellers (see Future Research for the multi-buyer CET game). Moreover, it is assumed that a buyer receives benefits from a coalition with sellers. If compliance enforcement is weak, self-interest buyers withdraw from the market without purchasing permits. However, political and economic benefits from international cooperation would encourage buyers to participate in the market (ancillary benefits of the IET [40, 57]). Under these assumptions, the CET game has a superadditive characteristic function. The collective payoff of the coalition, which is equal to the value of ancillary benefits for the buyer, is allocated to the members by Shapley value [45–47]. Shapley value gives a unique payoff allocation satisfying social rationality (Pareto efficiency). The solution also satisfies individual rationality in superadditive games. Shapley value reflects bargaining power of players (e.g. Shapley-Shubik power index [58]). Using Shapley value, I evaluate the supply-side and demand-side bargaining power (SBP and DBP). Permit price is derived from the buyer’s Shapley value, which is a function of bargaining power.

## Analysis

The CET game is a coalitional game with a buyer and *s*-sellers (*s* ≥ 1). Let **B**: = {−1} be the buyer set, and let **S**: = {1, 2,…, *s*} be the seller set. The set of players is **N**: = **B**∪**S**. Each player *i* ∈ **N** owns *C*_*i*_ (tCO_2_) of permits in advance. Assume *C*_−1_ < 0 for the buyer and *C*_*j*_ > 0 for any seller *j* ∈ **S**. A player can form a coalition with other players. A coalition *N* ⊆ **N** is a subset of the player set, and the CET game has 2^*s*+1^ coalitions. The coalitions are classified into four types. First, the empty coalition ∅ is the empty set. Second, the buyer coalition **B** is the buyer set. Third, a seller coalition *S*_+_ ⊆ **S** \ ∅ is a non-empty seller set. Fourth, a normal coalition **B**∪*S*_+_ contains the buyer and at least one seller. Permit demand and supply in **B**∪*S*_+_ are ∣*C*_−1_∣ and Γ_*S*_+__: = ∑_*j* ∈ *S*_+__ *C*_*j*_, respectively.

### Payoff Functions

A player’s payoff depends on the coalition to which the player belongs. The buyer’s payoff function is

$$\begin{array}{c} U_{-1}\left(N\right):=\{\begin{array}{cc} \left(\lambda -P\right)Q_{B\cup S_{+}} & \text{if}\;\;N=B\cup S_{+}\\ 0 & \text{otherwise} \end{array}. \end{array}$$ (1)

*P* is permit price per ton of CO_2_ emissions (*P* > 0). *Q*_**B**∪*S*_+__: = min{∣*C*_−1_∣, Γ_*S*_+__} is the volume of permits transferred from a seller coalition *S*_+_ to the buyer. The buyer’s willingness-to-pay (WTP), denoted by *λ*, is the maximum permit price that the buyer is willing to pay (*λ* ≥ 0). If a normal coalition **B**∪*S*_+_ is formed, the buyer pays *PQ*_**B**∪*S*_+__ for permits, and receives *λQ*_**B**∪*S*_+__ from the coalition. *λQ*_**B**∪*S*_+__ is interpreted as the value of the coalition for the buyer. The *j*-th seller’s payoff function is

$$\begin{array}{c} U_{j}\left(N\right):=\{\begin{array}{cc} Pq_{j} & \text{if}\;\;N=B\cup S_{+}\;\;\text{and}\;\;j\in S_{+}\\ 0 & \text{otherwise} \end{array}. \end{array}$$ (2)

*q*_*j*_ is the volume of permits transferred from seller *j* to the buyer, which satisfies ∑_*j* ∈ *S*_+__ *q*_*j*_ = *Q*_**B**∪*S*_+__.

### Characteristic Function

The collective payoff of a coalition is given by the characteristic function

$$\begin{array}{c} v\left(N\right):=\sum_{i\in N}U_{i}\left(N\right). \end{array}$$ (3)

The collective payoff can be positive only in normal coalitions (Table 1). *v* is a superadditive function which satisfies *v*(*N*_0_) + *v*(*N*_1_) ≤ *v*(*N*_0_ ∪ *N*_1_) for any two coalitions *N*_0_ and *N*_1_ such that *N*_0_ ∩ *N*_1_ = ∅. The collective payoff monotonically increases as the coalition size ∣*N*∣ increases. Hence the grand coalition **N** is expected to be formed. The CET game is the problem of how to allocate *v*(**N**) = *λQ*_**N**_ to players.

**Table 1:** Characteristic function *v*.

Type	Coalition *N*	Collective payoff *v*(*N*)
Empty	∅	0
Buyer	**B**	0
Seller	*S*_+_ ⊆ S \ ∅	0
Normal	**B** ∪ *S*_+_	*λQ*_**B**∪*S*_+__

The grand coalition is sustainable only if every player receives a non-negative payoff from it. If the payoff allocation to player *i* is negative, the player withdraws from the grand coalition and gains a zero payoff from the single coalition {*i*}. From *U*_−1_(**N**) ≥ 0 and *P* > 0, we obtain the condition for individual rationality:

$$\begin{array}{c} 0<P\leq \lambda . \end{array}$$ (4)

Only if the buyer has a positive WTP, permit price can be positive, and every player receives a non-negative payoff. If the buyer has a zero WTP, the grand coalition collapses, and every player receives a zero payoff.

**Proposition 1** *Permit price can be positive only if there exists a buyer with a positive WTP in the market*.

### Shapley Value

Shapley value gives a unique payoff allocation which satisfies social rationality (Pareto efficiency) [45–47]. As the characteristic function *v* is superadditive, Shapley value of the CET game also satisfies individual rationality (Eq (4)).

Let **M** be the set of (*s* + 1)! permutations of (*s* + 1) players. A permutation

m=(m(1),m(2),…,m(k),…,m(s+1))∈M (5)

indicates the order in which each player joins a coalition. Player *m*(1) forms the single coalition {*m*(1)}. Player *m*(2) forms the two-player coalition {*m*(1), *m*(2)} by joining {*m*(1)}. Player *m*(*k*) forms the *k*-player coalition {*m*(1), *m*(2),…, *m*(*k*)}. The coalition *N* ^*m*, *m*(*k*)^: = {*m*(1), *m*(2),…, *m*(*k* − 1)} is called the preceding coalition for player *m*(*k*). Due to superadditivity, the participation of player *i* in the preceding coalition *N* ^*m*, *i*^ monotonically increases the collective payoff. This payoff increase, measured by *v*(*N* ^*m*, *i*^∪{*i*}) − *v*(*N* ^*m*, *i*^), is the *i*-th player’s contribution in permutation *m*. Assume that every permutation occurs at the same probability. The *i*-th player’s Shapley value is the expected value of the contributions for all *m*:

$$\begin{array}{c} \phi_{i}:=\frac{\sum_{m\in M}\left(v\left(N^{m,i}\cup \left\{i\right\}\right)-v\left(N^{m,i}\right)\right)}{(s+1)!}. \end{array}$$ (6)

The buyer monotonically increases the collective payoff from zero to *λQ*_**B**∪*S*_+__ by joining a seller coalition *S*_+_ (Table 1). For each *S*_+_, there are ∣*S*_+_∣! (*s* − ∣*S*_+_∣)! permutations in which the buyer gains a non-zero contribution *λQ*_**B**∪*S*_+__. The buyer’s Shapley value is

$$\begin{array}{c} \phi_{-1}=\frac{\lambda \sum_{S_{+}\subseteq S\backslash \emptyset }|S_{+}|!(s-|S_{+}|)!Q_{B\cup^{\text{​}}S_{+}}}{\left(s+1\right)!}. \end{array}$$ (7)

Let *S*_(*j*)_ ⊆ **S** \ {*j*} be a seller coalition which does not contain seller *j*. *S*_(*j*)_ may be the empty coalition. The *j*-th seller gains a non-negative contribution *λ*(*Q*_*T*_(*j*)_∪{*j*}_ − *Q*_*T*_(*j*)__) by joining *T*_(*j*)_: = **B** ∪ *S*_(*j*)_. The *j*-th seller’s Shapley value is

$$\begin{array}{c} \phi_{j}=\frac{\lambda \sum_{S_{\left(j\right)}\subseteq S\backslash \left\{j\right\}}|T_{\left(j\right)}\left|!\;(s-\right|T_{\left(j\right)}\left|\right)!\;\left(Q_{T_{\left(j\right)}\cup \left\{j\right\}}-Q_{T_{\left(j\right)}}\right)}{(s+1)!}. \end{array}$$ (8)

The Shapley value satisfies social rationality

$$\begin{array}{c} \sum_{i\in N}\phi_{i}=v\left(N\right)=\lambda Q_{N}. \end{array}$$ (9)

The collective payoff of the grand coalition is allocated to the players with no loss. The Shapley value also satisfies individual rationality

$$\begin{array}{c} \phi_{i}\geq v\left(\left\{i\right\}\right)=0\;\;\text{for}\;\text{all}\;\;i\in N. \end{array}$$ (10)

Every player receives a non-negative payoff from the grand coalition.

### Permit Price

The buyer receives the Shapley value *ϕ*_−1_ from the grand coalition **N**. From *U*_−1_(**N**) = *ϕ*_−1_, we obtain permit price

$$\begin{array}{c} P=\lambda \left(1-\pi_{B}\right)=\lambda \pi_{S}, \end{array}$$ (11)

where

$$\begin{array}{c} \pi_{B}:=\frac{\phi_{-1}}{v(N)}\;\;\text{and}\;\;\pi_{S}:=\frac{\sum_{j\in S}\phi_{j}}{v(N)}. \end{array}$$ (12)

*π*_**B**_ ∈ [0, 1] and *π*_**S**_ ∈ [0, 1] are demand-side and supply-side bargaining power (DBP and SBP), respectively. The DBP is the proportion of the buyer’s Shapley value to the collective payoff. By social rationality (Eq (9)), *π*_**B**_ + *π*_**S**_ = 1. The DBP and SBP are independent of the buyer’s WTP.

**Proposition 2** *Permit price, which satisfies both individual and social rationality, is given by the product of the buyer’s WTP and the SBP (= 1 − DBP).*

### Range of Permit Price

Here I demonstrate that permit price ranges from *λ*/(*s* + 1) to *λ*/2. The DBP is written as

$$\begin{array}{c} \pi_{B}=\frac{\sum_{S_{+}}{\#}_{S_{+}}\rho_{S_{+}}}{(s+1)!}. \end{array}$$ (13)

#_*S*_+__: = ∣*S*_+_∣! (*s* − ∣*S*_+_∣)! is the number of player permutations in which a seller coalition *S*_+_ precedes the buyer. *ρ*_*S*_+__ is trading volume ratio (TVR) defined as

$$\begin{array}{c} \rho_{S_{+}}:=\frac{Q_{B\cup S_{+}}}{Q_{N}}=\frac{\min\{|C_{-1}|,\Gamma_{S_{+}}\}}{\min\{|C_{-1}|,\Gamma_{S}\}}. \end{array}$$ (14)

**Fig 3:**
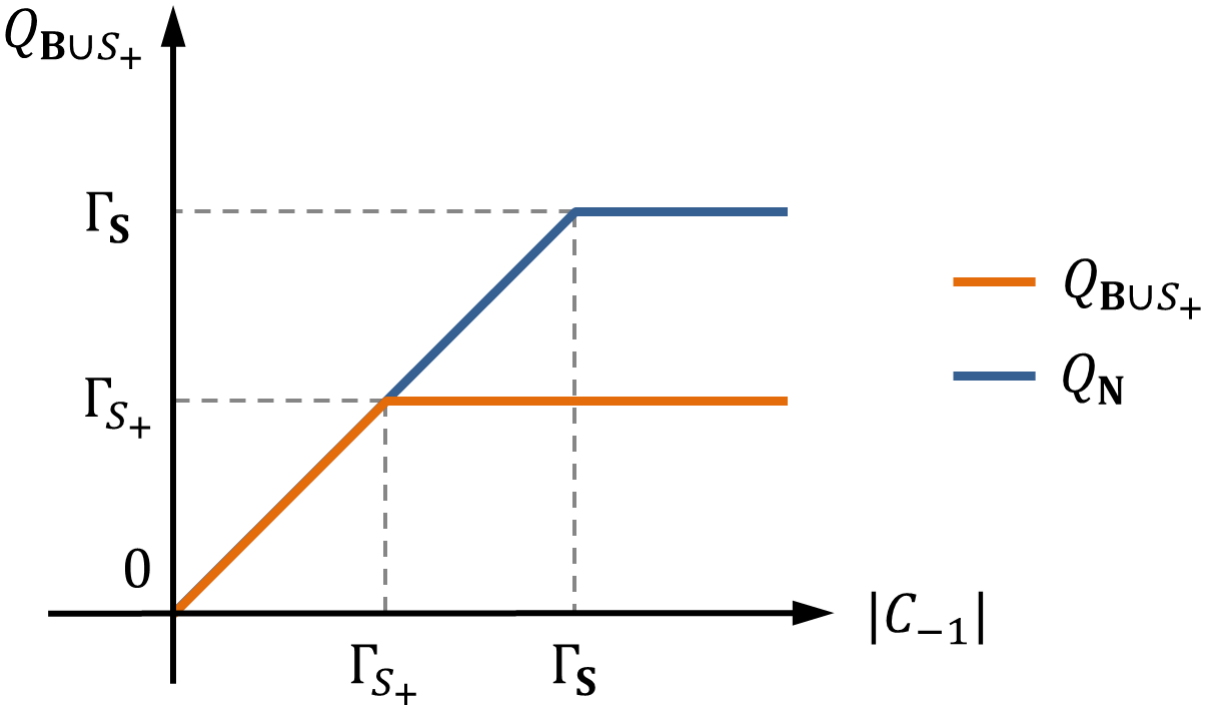
Trading volume of permits in a normal coalition **B** ∪ *S*_+_.

**Fig 4:**
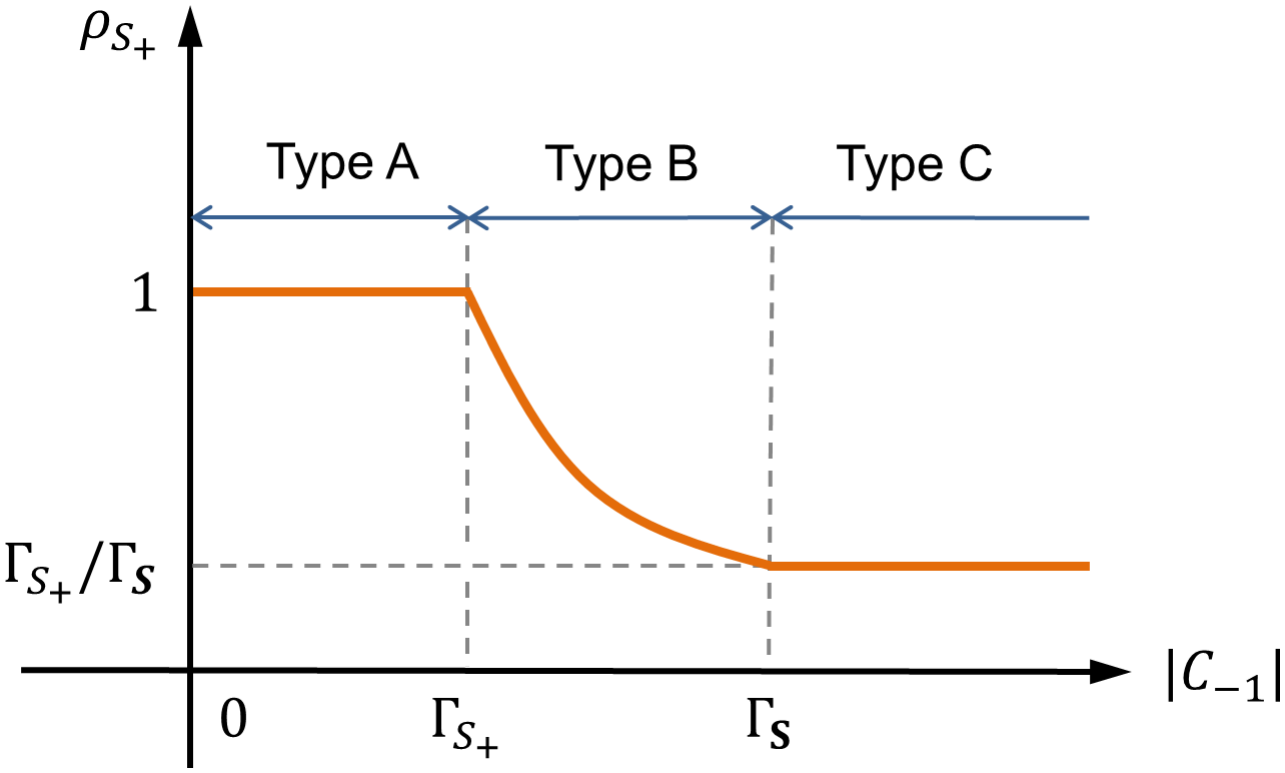
Trading volume ratio (TVR) in a normal coalition **B** ∪ *S*_+_.

Trading volume *Q*_**B**∪*S*_+__ is a piecewise linear and monotonically increasing function of permit demand ∣*C*_−1_∣ (Fig 3). Since Γ_*S*_+__ ≤ Γ_**S**_, the TVR is a monotonically decreasing function of ∣*C*_−1_∣ (Fig 4). Depending on the balance between supply and demand, the TVR is classified into three types: (A) ∣*C*_−1_∣/∣*C*_−1_∣, (B) Γ_*S*_+__/∣*C*_−1_∣, and (C) Γ_*S*_+__/Γ_**S**_.

#### Lower Limit of Permit Price

Suppose that the buyer’s WTP is constant. Permit price is minimized by maximizing the DBP (Eq (11)). The DBP is maximized if the TVR is type A for all *S*_+_. This condition is satisfied when permit supply is excessive, i.e., when ∣*C*_−1_∣ ≤ *C*_*j*_ for all seller *j*. Substituting *ρ*_*S*_+__ = 1 into Eq (13) gives the maximum DBP

$$\begin{array}{c} \pi_{B}^{\max}=\frac{\sum_{S_{+}}{\#}_{S_{+}}}{(s+1)!}. \end{array}$$ (15)

#_*S*_+__ is equal to the number of seller permutations in which *S*_+_ precedes **S** \ *S*_+_. Each seller permutation (*j*(1), *j*(2),…, *j*(*s*)) is generated from *s* preceding coalitions:

$$\begin{array}{c} \{j(1)\},\{j(1),j(2)\},\ldots ,\{j(1),j(2),\ldots ,j(s)\}. \end{array}$$ (16)

∑_*S*_+__ #_*S*_+__ counts every seller permutation *s* times. Hence ∑_*S*_+__ #_*S*_+__ = *s* × *s*!. The maximum DBP is

$$\begin{array}{c} \pi_{B}^{\text{max}}=\frac{s\times s!}{\left(s+1\right)!}=\frac{s}{s+1}. \end{array}$$ (17)

The lower limit of permit price is

$$\begin{array}{c} P^{\min}=\lambda \left(1-\pi_{B}^{\max}\right)=\frac{\lambda }{s+1}. \end{array}$$ (18)

#### Upper Limit of Permit Price

Permit price is maximized by minimizing the DBP. The DBP is minimized if the TVR is type C for all *S*_+_. This condition is satisfied when permit demand is excessive, i.e., when Γ_**S**_ ≤ ∣*C*_−1_∣. Substituting *ρ*_*S*_+__ = Γ_*S*_+__/Γ_**S**_ into Eq (13) gives the minimum DBP

$$\begin{array}{c} \pi_{B}^{\min}=\frac{\sum_{S_{+}}{\#}_{S_{+}}\Gamma_{S_{+}}}{\left(s+1\right)!\;\Gamma_{S}}=\frac{\sum_{S_{+}}\left({\#}_{S_{+}}\sum_{j\in S_{+}}C_{j}\right)}{\left(s+1\right)!\;\Gamma_{S}}. \end{array}$$ (19)

If *S*_+_ contains seller *j*, #_*S*_+__ *C*_*j*_ is added to the numerator of $\pi_{\text{B}}^{\text{min}}$. Let 𝓢_*j*_ be the set of seller coalitions containing seller *j*. Then

$$\begin{array}{c} \pi_{B}^{\min}=\frac{\sum_{j\in S}\alpha_{j}C_{j}}{\left(s+1\right)!\;\Gamma_{S}}, \end{array}$$ (20)

where *α*_*j*_: = ∑_*S*_+_ ∈ 𝓢_*j*__ #_*S*_+__. *α*_*j*_ should be same for all seller *j* because it is independent of permit supply and demand. We have

$$\begin{array}{c} \pi_{B}^{\min}=\frac{\alpha_{j}\Gamma_{S}}{\left(s+1\right)!\;\Gamma_{S}}=\frac{\alpha_{j}}{(s+1)!}. \end{array}$$ (21)

*α*_*j*_ is calculated as follows. A player permutation is written as Eq (5). If *S*_+_ is the single coalition {*j*}, seller *j* is *m*(1), the buyer is *m*(2), and (*s* − 1) sellers follow the buyer. The number of permutations is (*s* − 1)!. If *S*_+_ is a two-seller coalition containing seller *j*, seller *j* chooses *m*(1) or *m*(2), and another seller contained by *S*_+_ takes the remaining position. The buyer is *m*(3), and (*s* − 2) sellers follow the buyer. The number of permutations is 2(*s* − 1)!. If *S*_+_ is a *k*-seller coalition containing seller *j*, the number of permutations is *k*(*s* − 1)!. *α*_*j*_ is the sum of *k*(*s* − 1)! for all *k* ∈ {1,2,…, *s*}, which is

$$\begin{array}{c} \alpha_{j}=\left(1+2+\cdots +s\right)\left(s-1\right)!=\frac{(s+1)!}{2}. \end{array}$$ (22)

The minimum DBP is

$$\begin{array}{c} \pi_{B}^{\min}=\frac{1}{2}. \end{array}$$ (23)

The upper limit of permit price is

$$\begin{array}{c} P^{\max}=\lambda \left(1-\pi_{B}^{\min}\right)=\frac{\lambda }{2}. \end{array}$$ (24)

**Proposition 3** *Let *s* be the number of sellers. The DBP ranges from 1/2 to *s*/(*s* + 1). The DBP is always greater than or equal to the SBP.*

**Proposition 4** *Let *λ* be the buyer’s WTP. Permit price ranges from *λ*/(*s* + 1) to *λ*/2. Permit price is minimized when permit supply from every seller is greater than or equal to permit demand, and is maximized when permit demand is greater than or equal to the aggregate permit supply.*

### Monotonicity of Permit Price

Assume that permit supply and demand are independent of each other. Moreover, assume that the buyer’s WTP is constant. Under these assumptions, permit price satisfies three types of monotonicity: (I) Permit price monotonically increases as permit demand increases. (II) Permit price monotonically decreases as permit supply from a seller increases. (III) Permit price monotonically decreases with the entry of a new seller into the market.

#### Type I Monotonicity

Differentiating Eq (11) with respect to permit demand ∣*C*_−1_∣ gives

$$\begin{array}{c} \frac{\partial P}{\partial |C_{-1}|}=\left(1-\pi_{B}\right)\frac{\partial \lambda }{\partial |C_{-1}|}-\lambda \frac{\partial \pi_{B}}{\partial |C_{-1}|}. \end{array}$$ (25)

Since ∂*λ*/∂∣*C*_−1_∣ = 0, the type I monotonicity holds if ∂*π*_**B**_/∂∣*C*_−1_∣ ≤ 0.

The TVR (*ρ*_*S*_+__) is continuous over ∣*C*_−1_∣ > 0, and is differentiable almost everywhere. ∂*ρ*_*S*_+__/∂∣*C*_−1_∣ is non-positive for all types of the TVRs (Table 2). From Eq (13),

$$\begin{array}{c} \frac{\partial \pi_{B}}{\partial |C_{-1}|}=\frac{1}{(s+1)!}\sum_{S_{+}}{\#}_{S_{+}}\frac{\partial \rho_{S_{+}}}{\partial |C_{-1}|}\leq 0. \end{array}$$ (26)

Thus permit price satisfies the type I monotonicity.

**Table 2:** Derivative of the TVR with respect to ∣*C*_−1_∣.

Type	TVR	Condition	Derivative
A	∣*C*_−1_∣/∣*C*_−1_∣	∣*C*_−1_∣ ∈ (0, Γ_*S*_+__)	0
B	Γ_*S*_+__/∣*C*_−1_∣	∣*C*_−1_∣ ∈ (Γ_*S*_+__, Γ_**S**_)	< 0
C	Γ_*S*_+__/Γ_**S**_	∣*C*_−1_∣ ∈ (Γ_**S**_, ∞)	0

**Proposition 5**. *Assume that permit supply and demand are independent of each other. If the buyer’s WTP is constant, permit price monotonically increases as permit demand increases.*

#### Type II Monotonicity

Differentiating Eq (11) with respect to permit supply *C*_*j*_ gives

$$\begin{array}{c} \frac{\partial P}{\partial C_{j}}=\left(1-\pi_{B}\right)\frac{\partial \lambda }{\partial C_{j}}-\lambda \frac{\partial \pi_{B}}{\partial C_{j}}. \end{array}$$ (27)

Since ∂*λ*/∂*C*_*j*_ = 0, the type II monotonicity holds if ∂*π*_**B**_/∂*C*_*j*_ ≥ 0.

If *S*_+_ contains seller *j*, the TVR is

$$\begin{array}{c} \rho_{S_{+}}=\frac{\min\{|C_{-1}|,C_{j}+\Gamma_{S_{+}-\left\{j\right\}}\}}{\min\{|C_{-1}|,C_{j}+\Gamma_{S-\{j\}}\}}. \end{array}$$ (28)

The numerator and denominator are piecewise linear and monotonically increasing functions of *C*_*j*_. The TVR is continuous over *C*_*j*_ > 0, and is differentiable almost everywhere. ∂*ρ*_*S*_+__/∂*C*_*j*_ is non-negative for all types of the TVRs (Table 3). If *S*_+_ does not contain seller *j*, the TVR is

$$\begin{array}{c} \rho_{S_{+}}=\frac{\min\{|C_{-1}|,\Gamma_{S_{+}}\}}{\min\{|C_{-1}|,C_{j}+\Gamma_{S-\{j\}}\}}. \end{array}$$ (29)

The numerator is constant, and the denominator is same as Eq (28). ∂*ρ*_*S*_+__/∂*C*_*j*_ is zero for the types A and B, but is negative for the type C (Table 4).

**Table 3:** Derivative of the TVR with respect to *C*_*j*_ (*j* ∈ *S*_+_).

Type	TVR	Condition	Derivative
A	∣*C*_−1_∣/∣*C*_−1_∣	∣*C*_−1_∣ ∈ (0, Γ_*S*_+__)	0
B	(*C*_*j*_ + Γ_*S*_+_ \ {*j*}_)/∣*C*_−1_∣	∣*C*_−1_∣ ∈ (Γ_*S*_+__, Γ_**S**_)	> 0
C	(*C*_*j*_ + Γ_*S*_+_ \ {*j*}_)/(*C*_*j*_ + Γ_**S** \ {*j*}_)	∣*C*_−1_∣ ∈ (Γ_**S**_, ∞)	≥ 0

**Table 4:** Derivative of the TVR with respect to *C*_*j*_ (*j* ∉ *S*_+_).

Type	TVR	Condition	Derivative
A	∣*C*_−1_∣/∣*C*_−1_∣	∣*C*_−1_∣ ∈ (0, Γ_*S*_+__)	0
B	Γ_*S*_+__/∣*C*_−1_∣	∣*C*_−1_∣ ∈ (Γ_*S*_+__, Γ_**S**_)	0
C	Γ_*S*_+__/(*C*_*j*_ + Γ_**S** \ {*j*}_)	∣*C*_−1_∣ ∈ (Γ_**S**_, ∞)	< 0

If the TVR is type A or B for all *S*_+_, we obtain ∂*π*_**B**_/∂*C*_*j*_ ≥ 0 from Eq (13). If ∣*C*_−1_∣ ≥ Γ_**S**_, the TVR is type C for all *S*_+_, and the DBP has the minimum value of 1/2 (Proposition 3). Hence ∂*π*_**B**_/∂*C*_*j*_ = 0 if there exists *S*_+_ such that the TVR is type C. ∂*π*_**B**_/∂*C*_*j*_ is non-negative for all *S*_+_, and permit price satisfies the type II monotonicity.

**Proposition 6** *Assume that permit supply and demand are independent of each other. If the buyer’s WTP is constant, permit price monotonically decreases as permit supply from a seller increases.*

#### Type III Monotonicity

First, we expand the seller set **S** to **Z**: = **S** ∪ {*σ*} by adding a new seller *σ* with zero permit supply (Γ_{*σ*}_ = 0). The buyer forms the grand coalition with **Z**. Similar to Eq (13), the DBP is

$$\begin{array}{c} \pi_{B}^{*}=\frac{\sum_{Z_{+}\subseteq Z\backslash \emptyset }{\#}_{Z_{+}}^{*}\rho_{Z_{+}}^{*}}{\left(s+2\right)!}. \end{array}$$ (30)

*Z*_+_ is a non-empty seller coalition. ${\#}_{Z_{+}}^{*}:=|Z_{+}|!\;(s+1-|Z_{+}|)!$ is the number of player permutations in which *Z*_+_ precedes the buyer. $\rho_{Z_{+}}^{*}:=Q_{\text{B}\cup Z_{+}}/Q_{\text{B}\cup \text{Z}}$ is the TVR. Eq (11) gives permit price $P^{*}=\lambda (1-\pi_{\text{B}}^{*})$.

If *Z*_+_ = {*σ*}, *Q*_**B**∪*Z*_+__ = min{∣*C*_−1_∣, Γ_*Z*_+__} = 0 and $\rho_{Z_{+}}^{*}=0$. Hence ${\#}_{\left\{\sigma \right\}}^{*}\rho_{\left\{\sigma \right\}}^{*}=0$. If *Z*_+_ ≠ {*σ*}, *Z*_+_ is *S*_+_ or *S*_+_ ∪ {*σ*}. For every *S*_+_, *Q*_**B**∪*S*_+__ = *Q*_**B**∪*S*_+_∪{*σ*}_ and $\rho_{S_{+}}=\rho_{S_{+}}^{*}=\rho_{S_{+}\cup \{\sigma \}}^{*}$. Moreover,

$$\begin{array}{c} \begin{array}{cc} {\#}_{S_{+}}^{*}+{\#}_{S_{+}\cup \left\{\sigma \right\}}^{*} & =|S_{+}\left|!\;(s+1-\right|S_{+}|)!+\left(\right|S_{+}\left|+1)!\;(s-\right|S_{+}\left|\right)!\\ & =\left(s+2\right)|S_{+}\left|!\;(s-\right|S_{+}\left|\right)!\\ & =\left(s+2\right){\#}_{S_{+}}. \end{array} \end{array}$$ (31)

From Eq (30),

$$\begin{array}{c} \begin{array}{cc} \pi_{B}^{*} & =\frac{{\#}_{\left\{\sigma \right\}}^{*}\rho_{\left\{\sigma \right\}}^{*}+\sum_{S_{+}}\left({\#}_{S_{+}}^{*}\rho_{S_{+}}^{*}+{\#}_{S_{+}\cup \left\{\sigma \right\}}^{*}\rho_{S_{+}\cup \left\{\sigma \right\}}^{*}\right)}{(s+2)!}\\ & =\frac{\sum_{S_{+}}\left({\#}_{S_{+}}^{*}+{\#}_{S_{+}\cup \left\{\sigma \right\}}^{*}\right)\rho_{S_{+}}}{(s+2)!}\\ & =\frac{\left(s+2\right)\sum_{S_{+}}{\#}_{S_{+}}\rho_{S_{+}}}{(s+2)!}\\ & =\pi_{B}, \end{array} \end{array}$$ (32)

and we obtain P* = *P*. The entry of seller *σ* into the market has no influence on permit price.

Second, we increase permit supply from seller *σ* to an arbitrary level. By Proposition 6, this operation monotonically decreases permit price. Now we find that permit price satisfies the type III monotonicity.

**Proposition 7** *Assume that permit supply and demand are independent of each other. If the buyer’s WTP is constant, permit price monotonically decreases with the entry of a new seller into the market.*

### Results and Discussion

The seven propositions derived from the CET game help us understand why permit buyers could have dominant power. This section summarizes the results of the CET game based on market data. Stability of coalitions is also evaluated. Finally, limitations and extensions of the CET game are discussed.

#### Withdrawal of Buyers from the International Permit Market

In the CET game, a buyer purchases permits from sellers only if the buyer forms a normal coalition with the sellers. Whether a normal coalition is formed or not depends on the buyer’s WTP. A buyer with a zero WTP withdraws from the market without purchasing permits. Only if there exists a buyer with a positive WTP, permit price can be positive (Proposition 1). The permit price, which satisfies both individual and social rationality, is given by the product of the buyer’s WTP and the SBP (Proposition 2). Permit price is strongly influenced by the buyer’s WTP.

Buyers can withdraw from the Kyoto Protocol without paying costs. The IET under CP1 experienced the withdrawal of the top-three buyers: the US, Japan, and Canada. The US, which is the largest buyer in Annex B Parties, did not ratify the Kyoto Protocol. The US is a buyer with a zero WTP. Japan has purchased a large amount of permits from transition economies such as Ukraine, Czech, Latvia, and Poland [59], but decided not to participate in the second commitment period (CP2) [22, 23]. After the severe nuclear disaster in Fukushima, Japan abandoned the 2010 Basic Energy Plan in which the promotion of nuclear power generation was emphasized [60]. As of February 2015, all nuclear power plants of Japan are closed for safety reasons, and most of the electricity is supplied from fossil fuel power plants [61]. The increasing dependence on fossil fuels leads to substantial increases in CO_2_ emissions [62]. The costs for additional permits, which are expected to be huge under CP2, may have decreased Japan’s WTP to zero. Canada also withdrew from the Kyoto Protocol to avoid financial burdens associated with the IET [21, 23]. The withdrawal of the top-three buyers indicates that the buyer’s WTP is sensitive to changes in political and economic conditions.

The right of withdrawal is a threat to sellers. Unless a buyer with a positive WTP joins the coalition, sellers cannot gain benefits from emissions trading. Therefore the buyer’s contribution to the coalition is relatively large, and sellers are forced to suppress permit price to less than *λ*/2 (Proposition 4). This result is equivalent to Proposition 3 that the DBP is always greater than or equal to the SBP. Sellers cannot dominate the market even if permit demand is excessive.

#### Excessive Permit Supply from Transition Economies

**Fig 5:**
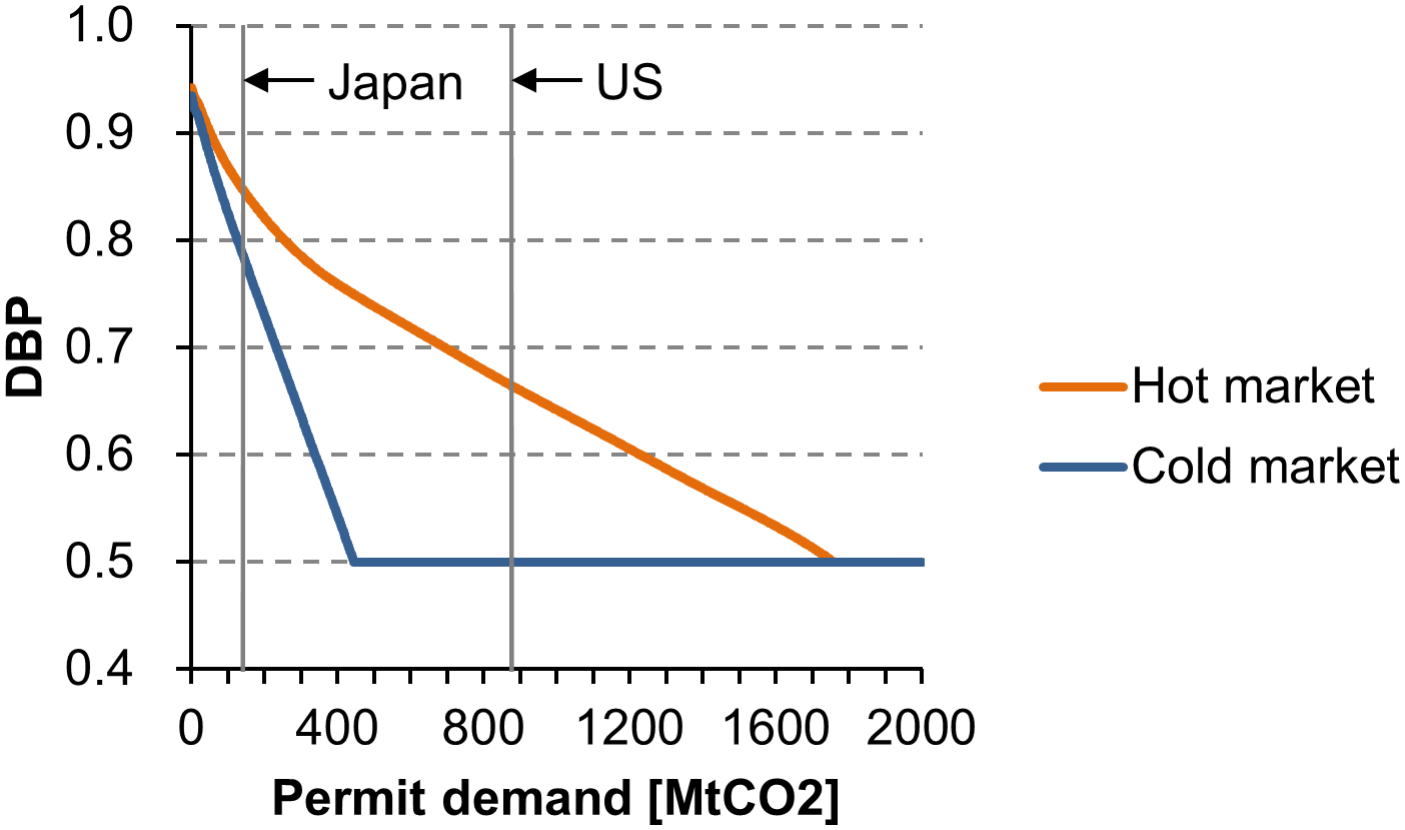
Curves of the demand-side bargaining power (DBP) in hot and cold markets. The DBP is obtained by putting the number of sellers (*s*), supply data (*C*_1_, *C*_2_,…, *C*_*s*_), and demand data (∣*C*_−1_∣) in Eq (12). Both markets contain 18 sellers (*s* = 18; Belgium, Bulgaria, Croatia, Czech Republic, Denmark, Estonia, Germany, Hungary, Latvia, Lithuania, Monaco, Poland, Romania, Russian Federation, Slovakia, Sweden, Ukraine, and the UK). The hot market is the real market shown by Fig 1. In the cold market, the supply from Russia and Ukraine is assumed to be zero. Each curve consists of 2,000 points of the DBPs corresponding to ∣*C*_−1_∣ = 1,2,…,2000 (MtCO_2_).

The excessive permit supply from transition economies (hot air) increases the DBP and decreases permit price. Suppose that permit supply and demand are independent of each other, and that the buyer’s WTP is constant. Permit price monotonically decreases as the supply from a seller increases (Proposition 6). A decrease in permit price means an increase in the DBP (Proposition 2). Fig 5 compares the DBP curves in two types of markets: hot and cold. Both markets contain 18 sellers (*s* = 18). The hot market is the real market shown by Fig 1. In the cold market, the supply from Russia and Ukraine is assumed to be zero. In both markets, the DBP monotonically decreases as the demand increases (Proposition 5). The DBP in the hot market is greater than or equal to the DBP in the cold market. For instance, Japan’s DBP is 0.848 in the hot market, and is 0.786 in the cold market. The supply from Russia and Ukraine enables Japan to purchase permits at a 29% discount (Eq (11)). The hot air reinforces the demand-side dominance in the international permit market.

As discussed by many authors [25–30], the absence of the US from the market has accelerated the downward trend in permit price. The DBP of the US is 0.664 in the hot market (Fig 5). If the US has the same WTP as Japan, permit price for the US is 120% higher than that for Japan (Eq (11)). From this result, we immediately find that the withdrawal of Japan and Canada from the market decreases permit price. Buyers with smaller demands have higher DBPs, and purchase permits at lower prices. Meanwhile, Russia decided not to participate in CP2 [23]. The withdrawal of Russia means a substantial decrease in the aggregate permit supply, which may bring upward pressure on permit price (Proposition 7).

#### Market Power of Seller Cartels

**Fig 6:**
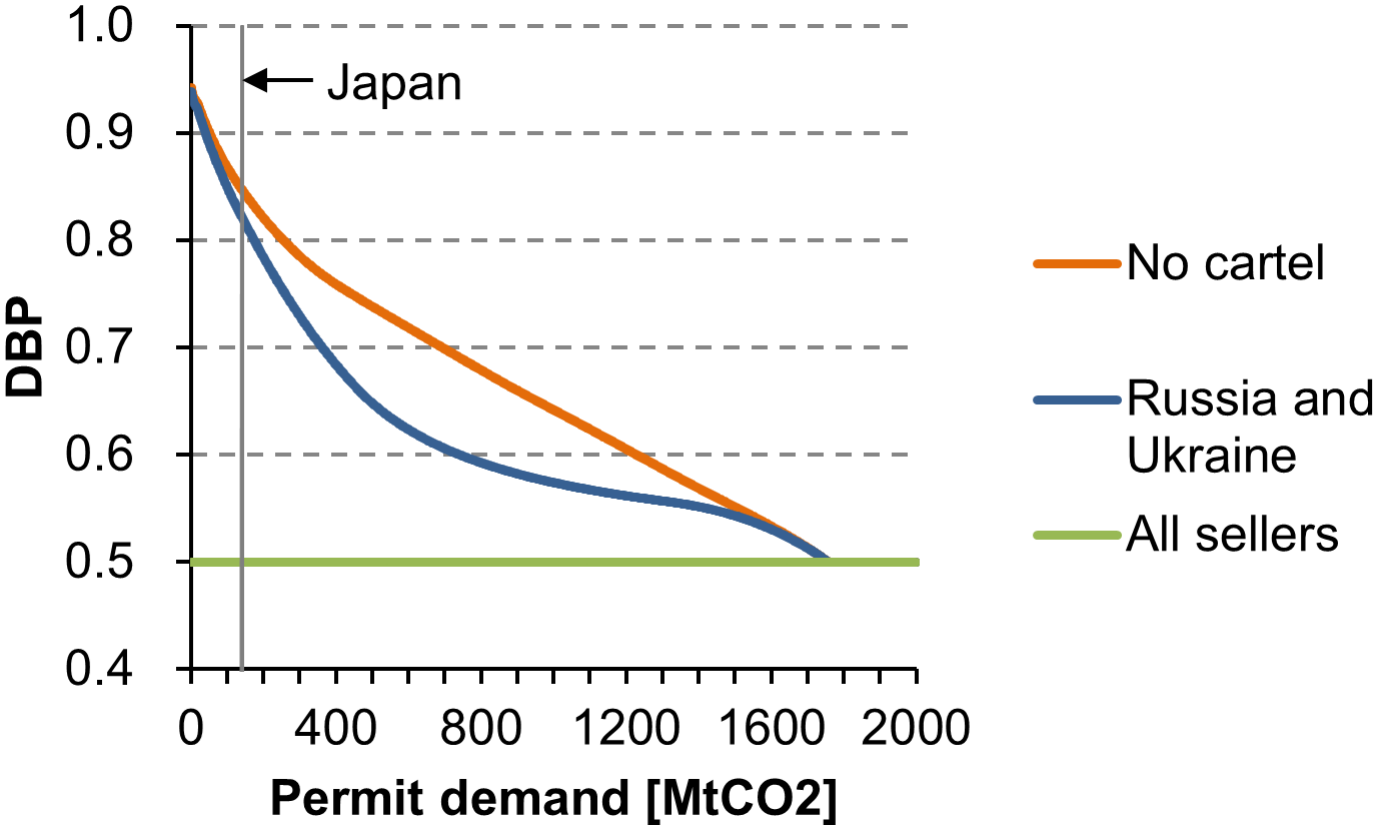
Impacts of seller cartels on the DBP. Calculated from the data of Fig 1. The cartel of Russia and Ukraine controls 75% of the aggregate permit supply.

Previous studies conclude that transition economies can increase permit price by forming seller cartels [26–28, 30, 33]. However, the demand-side dominance resists market power of seller cartels. Fig 6 shows the DBP curves under no cartel, the cartel of Russia and Ukraine, and the cartel of all sellers. The cartel of Russia and Ukraine controls 75% of the aggregate permit supply. If sellers form a cartel, the sellers withdraw from the market, which decreases the DBP (Proposition 7). At the same time, the seller cartel with a large permit supply enters the market, which increases the DBP. The cartel of Russia and Ukraine increases the SBP (decreases the DBP). For instance, Japan’s DBP decreases from 0.848 (under no cartel) to 0.822, which implies a 17% increase in permit price (Eq (11)). The SBP achieves the maximum value of 0.5 when all sellers form the grand cartel (*s* = 1, Proposition 3). In this case, the SBP is equal to the DBP. The formation of seller cartels weakens the demand-side dominance, but cannot reverse it.

#### Impacts of the Global Financial Crisis

**Fig 7:**
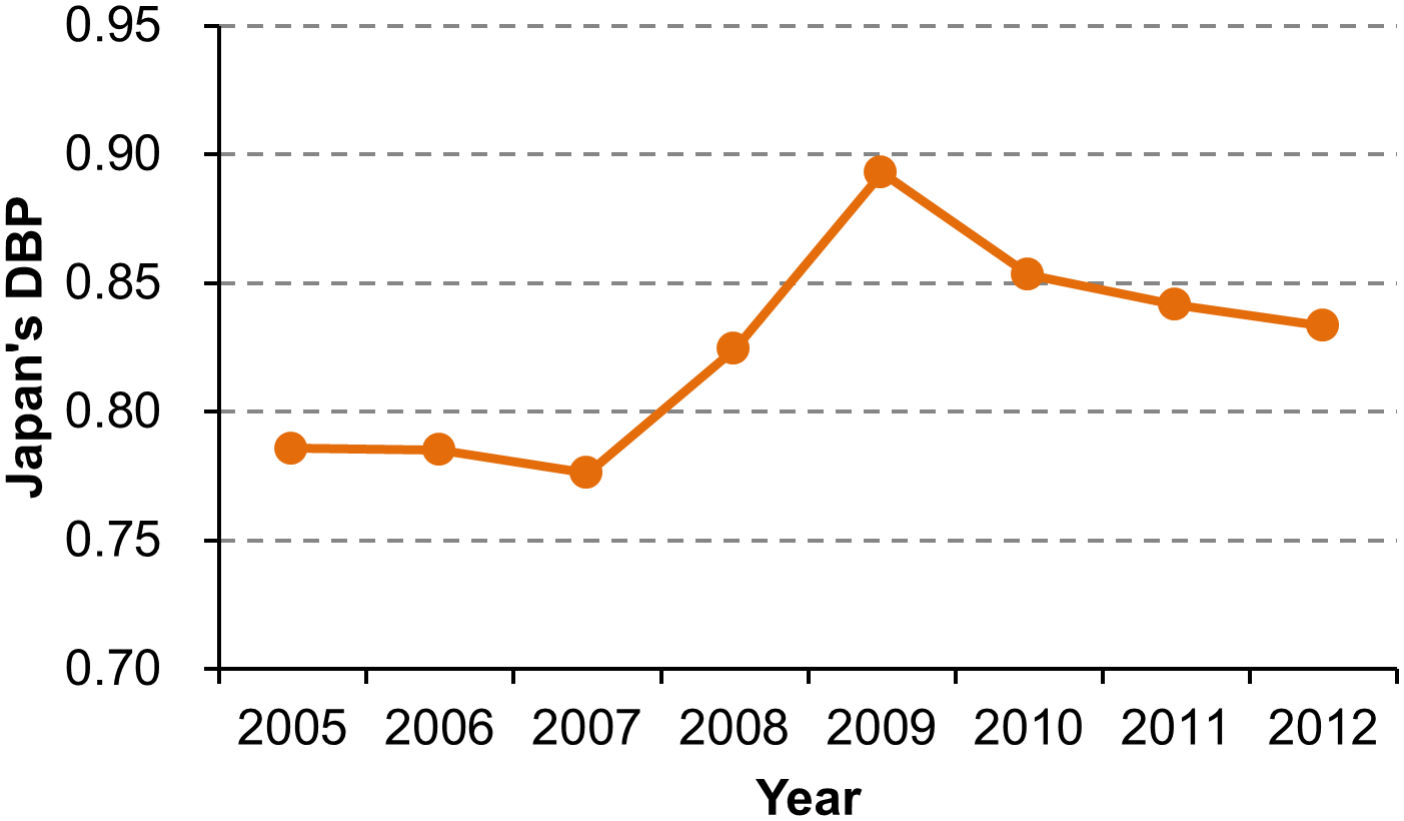
Japan’s DBP between 2005 and 2012. Calculated from the data of Fig 1.

The 2008–2009 global financial crisis slowed down economic growth in Annex B Parties. Due to the economic downturn, CO_2_ emissions from the Parties except the US decreased from 8,382 MtCO_2_ in 2008 to 7,803 MtCO_2_ in 2009 [24]. The Kyoto Protocol has no mechanism to adjust the balance between permit supply and demand. As the permit allocation to each country is fixed, the decrease in CO_2_ emissions increased the supply from transition countries, and decreased the demand of Japan, Canada, and other buyers (Fig 2). By Propositions 5 and 6, permit price monotonically decreases as permit supply (demand) increases (decreases). Fig 7 shows Japan’s DBP between 2005 and 2012. Japan’s DBP increased from 0.776 in 2007 to 0.893 in 2009, which means a 52% decrease in permit price (Eq (11)). This result suggests that the global financial crisis contributed to the demand-side dominance.

In the real market, AAU price responded to the global financial crisis with a time lag. The annual average price of AAUs peaked in 2009, and plunged in 2011 [12–14]. This time lag is attributed to a time-consuming process of international bargaining. In March 2009, Japan and Ukraine concluded a contract for the permit transfer of 30 MtCO_2_ after spending eight months in bargaining [63]. Another contract for the permit transfer of 40 MtCO_2_ from Czech to Japan, which was concluded in the same month, required six months of bargaining [64]. The AAU prices specified in these contracts reflect market conditions before the recession.

#### Stability of Grand Coalitions

The CET game considers bargaining in single-buyer grand coalitions (SBGCs). Annex B Parties have 19 permit buyers except for the US, and hence the number of possible SBGCs is 19. It is expected that the most stable SBGC would be chosen by sellers. The stability of coalitions is measured by stability index (SI) [49, 65–68]. The SI is the coefficient of variation of players’ power indices (PIs). The PI is a similar value to the Shapley-Shubik power index [48, 58]. In the CET game, the PI is equivalent to bargaining power (Eq (12)):

$$\begin{array}{c} {\text{PI}}_{i}:=\frac{U_{i}\left(N\right)-v\left(\left\{i\right\}\right)}{\sum_{j\in N}\left(U_{j}\left(N\right)-v\left(\left\{j\right\}\right)\right)}=\frac{U_{i}\left(N\right)}{v(N)}=\frac{\phi_{i}}{v(N)}=\pi_{i}. \end{array}$$ (33)

A coalition with a lower SI is more stable. A coalition is completely stable (SI = 0) when all players have the same PI.

Table 5 lists the PIs of Japan and all sellers in the grand coalition. Japan is a dominant buyer with high bargaining power (see also Fig 5). In sellers, Russia, Ukraine, and Germany have relatively high PIs. Meanwhile, Croatia, Belgium, and Monaco cannot exercise bargaining power. Each player’s payoff is equal to the product of the PI and the collective payoff (*ϕ*_*i*_ = *π*_*i*_ × *v*(**N**)). Japan receives 85% of the collective payoff, and the top-three sellers receive 51% of the permit sales. Table 6 lists the SIs of the SBGCs. Due to the demand-side dominance, all the coalitions have high SIs. In Japan’s coalition, for instance, the standard deviation of the PIs is more than three times higher than the mean PI (0.053). This result suggests that a SBGC is generally unstable. A SBGC with a larger buyer has a higher SI. The US coalition has the lowest SI, but the US is absent from the market. Sellers are expected to join Japan’s coalition, which is the most stable coalition next to the US coalition.

**Table 5:** Power indices (PIs) of Japan and all sellers in the grand coalition.

Country	PI
Japan	0.848
Russia	0.026
Ukraine	0.026
Germany	0.026
Romania	0.019
UK	0.012
Czech	0.009
Poland	0.007
Bulgaria	0.006
Slovakia	0.005
Lithuania	0.005
Hungary	0.004
Estonia	0.004
Latvia	0.002
Sweden	0.001
Denmark	0.001
Croatia	0.000
Belgium	0.000
Monaco	0.000

Calculated from the data of Fig 1.

**Table 6:** Stability indices (SIs) of single-buyer grand coalitions (SBGCs).

Buyer	SI
US	2.844
Japan	3.564
Canada	3.596
Australia	3.653
Spain	3.697
Netherlands	3.889
Greece	3.901
Italy	3.909
France	3.927
Austria	3.930
Portugal	3.931
Ireland	3.934
New Zealand	3.940
Norway	3.940
Finland	3.956
Switzerland	3.964
Slovenia	3.966
Iceland	3.982
Luxembourg	3.997
Lichtenstein	4.007

Calculated from the data of Fig 1.

### Future Research

The CET game uses three assumptions to simplify the calculation of bargaining power. First, players form the grand coalition. This assumption is popular in cooperative games, but there is no strong evidence. In the real world, a large coalition is not necessarily efficient because the complicated process of international bargaining costs the members. Moreover, a buyer’s WTP changes depending on sellers. There may be a pair of countries which cannot cooperate for political reasons. If these factors break superadditivity of the characteristic function, the grand coalition would divide into smaller coalitions. Results from non-cooperative games suggest that large coalitions are unstable in international climate negotiations [35–37, 39, 42]. A direction of future research is to couple the CET game with non-cooperative games which describe the formation of stable coalitions.

Second, multi-buyer coalitions are excluded. The extension of the buyer set makes it difficult to determine the payoff allocation. Let **B** = {−1, −2,…, −*b*} be the buyer set, and let **S** = {1, 2,…, *s*} be the seller set. Buyer *i* ∈ **B** has a demand of ∣*C*_*i*_∣ (*C*_*i*_ < 0), and seller *j* ∈ **S** has a supply of *C*_*j*_ (*C*_*j*_ > 0). Let *λ*_*i*_ be each buyer’s WTP. In a normal coalition *N*_+_ = *B*_+_ ∪ *S*_+_, trading volume of permits is given by *Q*_*N*_+__ = min{∑_*i* ∈ *B*_+__∣*C*_*i*_∣, ∑_*j* ∈ *S*_+__ *C*_*j*_}. The total payoff of all sellers is *PQ*_*N*_+__. Let *r*_*i*_ ∈ [0, ∣*C*_*i*_∣] be the volume of permits transferred from sellers to buyer *i* (∑_*i*_ *r*_*i*_ = *Q*_*N*_+__). Each buyer’s payoff function is (*λ*_*i*_ − *P*)*r*_*i*_, and the total payoff of all buyers is ∑_*i*_ *λ*_*i*_ *r*_*i*_ − *PQ*_*N*_+__. The collective payoff of the normal coalition is ∑_*i*_ *λ*_*i*_ *r*_*i*_. This characteristic function is not always superadditive (Table 7). In this example, we cannot expect the grand coalition. Even if the grand coalition is formed, the Shapley value does not satisfy individual rationality. A different approach is required to solve the multi-buyer CET game.

**Table 7:** Characteristic function of a multi-buyer CET game.

Coalition *N*	Collective payoff *v*(*N*)
*ϕ*	0
{−1}	0
{−2}	0
{1}	0
{−1, −2}	0
{−1, 1}	*λ*_−1_ *Q*_{−1, 1}_ = 10
{−2, 1}	*λ*_−2_ *Q*_{−2, 1}_ = 20
{−1, −2, 1}	*λ*_−1_ *r*_−1_ + *λ*_−2_ *r*_−2_ = 15

The following parameters are assumed: *b* = 2, *s* = 1, ∣*C*_−1_∣ = 10, ∣*C*_−2_∣ = 10, *C*_1_ = 10, *λ*_−1_ = 1, *λ*_−2_ = 2, *r*_−1_ = 5, and *r*_−2_ = 5. The participation of buyer −1 into the coalition {−2, 1} decreases the collective payoff. This characteristic function does not satisfy superadditivity.

Third, the CET game uses Shapley value to determine the payoff allocation. Shapley value is a popular solution concept for coalitional games, but various alternatives have been proposed (e.g. Nash-Harsanyi bargaining solution and nucleolus). For instance, Gately [69] proposed the solution based on propensity to disrupt (PTD). The *i*-th player’s PTD is defined as

$$\begin{array}{c} d_{i}:=\frac{\sum_{j\in N\backslash \left\{i\right\}}U_{j}\left(N\right)-v\left(N\backslash \left\{i\right\}\right)}{U_{i}\left(N\right)-v\left(\left\{i\right\}\right)}=\frac{v\left(N\right)-v\left(N\backslash \left\{i\right\}\right)}{U_{i}\left(N\right)}-1. \end{array}$$ (34)

A high PTD means that the participation of player *i* in the grand coalition is highly beneficial to other players but is not to the player [49, 65–67]. Gately solution is the payoff allocation $\left\{\phi_{i}^{\prime }|i\in \text{N}\right\}$ such that all players have the same PTD. Does the shift from Shapley value to Gately solution affect the results of the CET game?

I conclude this paper by comparing Gately solution and Shapley value in the three-player CET game **N** = {−1, 1,2}. From Gately solution, the buyer receives

$$\begin{array}{c} \phi_{-1}^{\prime }=\frac{\lambda Q_{N}^{2}}{3Q_{N}-Q_{\left\{-1,1\right\}}-Q_{\left\{-1,2\right\}}}. \end{array}$$ (35)

The Gately-DBP is

$$\begin{array}{c} \pi_{B}^{\prime }=\frac{\phi_{-1}^{\prime }}{v(N)}=\frac{Q_{N}}{3Q_{N}-Q_{\left\{-1,1\right\}}-Q_{\left\{-1,2\right\}}}, \end{array}$$ (36)

and the Shapley-DBP is

$$\begin{array}{c} \pi_{B}=\frac{\phi_{-1}}{v(N)}=\frac{2Q_{N}+Q_{\left\{-1,1\right\}}+Q_{\left\{-1,2\right\}}}{6Q_{N}}. \end{array}$$ (37)

The difference between the two DBPs is

$$\begin{array}{c} \pi_{B}^{\prime }-\pi_{B}=\frac{\left(Q_{\left\{-1,1\right\}}+Q_{\left\{-1,2\right\}}-Q_{N}\right)\left(Q_{\left\{-1,1\right\}}+Q_{\left\{-1,2\right\}}\right)}{6Q_{N}\left(3Q_{N}-Q_{\left\{-1,1\right\}}-Q_{\left\{-1,2\right\}}\right)}\geq 0. \end{array}$$ (38)

The Gately-DBP is greater than or equal to the Shapley-DBP. The shift from Shapley value to Gately solution reinforces the demand-side dominance. If ∣*C*_−1_∣ ≥ *C*_1_ + *C*_2_,

$$\begin{array}{c} \pi_{B}=\pi_{B}^{\prime }=\frac{1}{2}. \end{array}$$ (39)

Under the excessive permit demand, the Gately-DBP is equal to the Shapley-DBP. If ∣*C*_−1_∣ ≤ *C*_1_ and ∣*C*_−1_∣ ≤ *C*_2_,

$$\begin{array}{c} \pi_{B}=\frac{2}{3}\;\;\text{and}\;\;\pi_{B}^{\prime }=1. \end{array}$$ (40)

Gately solution gives the payoff allocation in which the buyer monopolizes the collective payoff. From $U_{-1}=\phi_{-1}^{\prime }$ (Eq (1)), permit price is zero. Under the excessive permit supply, Gately solution fails to provide a feasible payoff allocation.
